# A Chinese conundrum: does higher insurance coverage for hospitalization reduce financial protection for the patients who most need it?

**DOI:** 10.1093/heapol/czae108

**Published:** 2024-11-09

**Authors:** Xiaoying Zhu, Ajay Mahal, Shenglan Tang, Barbara McPake

**Affiliations:** Nossal Institute for Global Health, Melbourne School of Population and Global Health, The University of Melbourne, 32 Lincoln Square, Carlton, Victoria 3053, Australia; School of Elderly Care Services and Management, Nanjing University of Chinese Medicine, 138 Xianlin Rd, Nanjing, Jiangsu 210023, China; Nossal Institute for Global Health, Melbourne School of Population and Global Health, The University of Melbourne, 32 Lincoln Square, Carlton, Victoria 3053, Australia; Global Health Research Center, Duke Kunshan University, No. 8 Duke Avenue, Kunshan, Jiangsu 215316, China; Duke Global Health Institute, Duke University, 310 Trent Drive, Durham, NC 27710, USA; SingHealth Duke-NUS Global Health Institute, Duke-NUS, 8 College Road, Singapore 169857, Singapore; Nossal Institute for Global Health, Melbourne School of Population and Global Health, The University of Melbourne, 32 Lincoln Square, Carlton, Victoria 3053, Australia

**Keywords:** health insurance, healthcare utilization, cost-sharing, hospitalization, equity

## Abstract

This paper evaluates the relationship between the degree of cost-sharing and the utilization of outpatient and inpatient health services in China. Using data from the 2015 China Health and Retirement Longitudinal Study (CHARLS), we estimated the association between outpatient and inpatient service utilization and cost-sharing levels associated with outpatient and inpatient services, as well as a comparative metric that quantifies the relative cost-sharing burden between the two. We found that patients in areas with higher levels of cost-sharing for outpatient services exhibit a lower propensity to use outpatient care and a higher inclination to utilize costly hospitalization services. Conversely, as the ratio of cost-sharing for outpatient services to that for inpatient services increases, the likelihood of patients forgoing doctor-initiated hospitalization correspondingly increases. This suggests that when cost-sharing for outpatient care rises relative to inpatient care, observed increases in inpatient care utilization reflect an escalation in moral hazard rather than a correction for the underutilization of inpatient services. We conclude that both substitution and complementary roles exist between outpatient and inpatient services. Our findings suggest that a more effective design of cost-sharing is needed to enhance the equity and efficiency of China’s health system.

Key messages

- The observed increases in inpatient care utilization reflect an escalation in moral hazard rather than a correction for the underutilization of inpatient services.
- Higher cost-sharing for outpatient services nudges individuals towards unnecessary inpatient service use.
- Patients from rural areas, with chronic diseases, or with low incomes are more sensitive to the level of cost-sharing, thus tailored health insurance policies should be set up for these populations to ensure they have equitable access to health services.

## Introduction

As healthcare systems worldwide face rapid growth in health spending and varying demands, the role of health insurance has become increasingly critical in promoting individuals’ access to health services and protecting them against financial risks (Kellermann 2002). However, finding a balance between coverage and cost control is a complex challenge. Cost-sharing, which involves patients paying a portion of their healthcare costs, has been used as an important tool to contain healthcare costs and improve efficiency (Han et al. 2020). The idea behind cost-sharing is the belief that when patients bear a portion of their healthcare expenses, they become more conscientious about their healthcare utilization. This conscientiousness potentially reduces unnecessary visits or hospitalizations, thereby curtailing overall healthcare expenditures and contributing to more efficient use of healthcare resources. Many countries have incorporated cost-sharing schemes when designing their health insurance programmes. Evidence in different countries has shown that by imposing direct financial implications for healthcare utilization, cost-sharing indeed influences healthcare-seeking behaviour and service usage (Rice and Morrison 1994, Feng et al. 2020). However, it is important to critically assess the impact of cost-sharing on health service utilization. Research has shown that cost-sharing leads to decreased usage of nearly all health care, thereby achieving its primary aim of cost containment (Chalkley and Malcomson 2002, Chandra et al. 2014). However, in countries with higher health insurance protection levels, cost-sharing reduction triggers a ‘moral hazard effect’, where individuals may use more healthcare services than necessary, and the welfare loss from this moral hazard may outweigh the benefits provided by the health insurance (Pauly 1968). For example, Baicker and Goldman (Baicker and Goldman 2011) point out that although cost-sharing could potentially improve the efficiency of health service use, its lack of discrimination between high-value and low-value uses, as well as its failure to consider crucial interactions between cost mechanisms and patient behaviour, make it generally a blunt tool in its current application within most health insurance plans.

**Figure 1.**
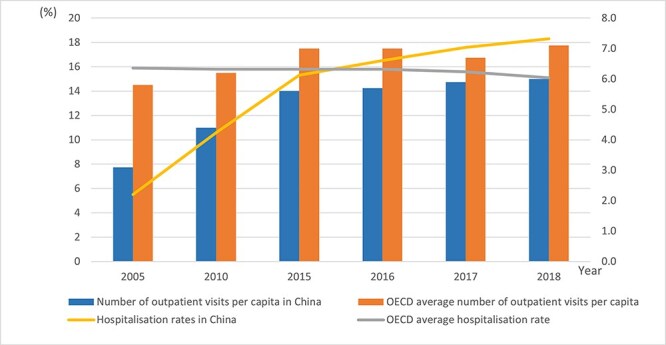
Outpatient and inpatient services utilization in China and OECD countries from 2005 to 2018. Source: 2019 China Health Statistical Yearbook; OECD Health Statistics 2020.

In the Chinese context, a key achievement is its almost universal population coverage by the government-led social health insurance (SHI) schemes (Yip et al. 2012, He et al. 2022). SHI is comprised of three primary health insurance schemes: Urban Employee Basic Medical Insurance (UEBMI), Urban Resident Basic Medical Insurance (URBMI), and New Rural Cooperative Medical Insurance (NRCMI). The latter two have been progressively merged across various regions into a unified scheme known as Urban and Rural Residents Basic Medical Insurance (URRBMI) since 2016. A key policy concern, subject to considerable debate (He and Shen 2018, Zhang 2021), has been the following: should the emphasis be on coverage for severe illnesses, which are mainly managed through inpatient services, or minor illnesses predominantly addressed through outpatient services? Given resource constraints and concerns about the incidence of catastrophic health expenditures experienced by the population, the initial emphasis on the design of insurance was on ensuring that inpatient expenditures were reimbursable. For outpatient expenses, including drugs, monthly reimbursements are capped at a relatively low rate, and patient shares of the total costs of inpatient care are typically lower than for outpatient care across provinces/regions (Shen et al. 2020). In the Chinese health system, a dual urban–rural structure is evident and cost-sharing rates for outpatient and inpatient services vary across regions and provinces. However, patients in both settings, across most provinces/regions are free to choose their healthcare providers and can directly seek treatment from any hospital. They do not require prior appointments to access these hospitals. Furthermore, primary care providers play a limited role in both serving as the initial point of contact and effectively coordinating with specialty care (Li et al. 2020). In this situation, differences in health service cost-sharing levels across inpatient and outpatient care might affect people seeking health services inefficiently. Available data suggests that the hospitalization rate of residents in China has been steadily rising. In 2018, it surpassed the average levels of Japan, the UK, and the Organization for Economic Cooperation and Development (OECD) countries (Fig. 1). However, outpatient visits per capita in China are the lowest among these countries.

International evidence has established that higher levels of cost-sharing are likely to deter individuals from seeking health care (Newhouse 1993) and highlights the propensity of cost-sharing to diminish the use of healthcare services deemed necessary and appropriate (Wong et al. 2001, Choudhry et al. 2010, Krack 2019, Fusco et al. 2023). Some studies have shown that changes in cost-sharing can affect the use of different types of services. For instance, Trivedi et al. (2010) analysed the benefits for Medicare plans in the USA and found that increased co-payments for ambulatory care curtailed outpatient visits but increased hospitalization and inpatient days. Chandra *et al*. (Chandra et al. 2014) investigated the effects of rising cost-sharing on low-income populations in the Massachusetts Commonwealth Care programme, suggesting that inpatient and outpatient services could substitute for each other. Kato and Goto (Kato and Goto 2017) utilized administrative hospital admissions data across regions in Japan and found that subsidies for outpatient care led to fewer overall hospital admissions in low-income regions, while the opposite trend was noted in more affluent areas. These findings suggest that the cross-price effect (or the effect of a change in the price of one service on the demand for the other) in healthcare service utilization might depend on contextual factors, such as the socio-economic status of the population studied.

While there is an extensive literature examining the relationship between health insurance generosity and healthcare utilization, evidence on how changes in cost-sharing affect the use of inpatient and outpatient services in China is limited. Also, evidence comparing the moral hazard effect and the demand release effect is scarce. He (2022) examined the impact of implementing a financing scheme for outpatient care on inpatient utilization and expenditure within China’s UEBMI scheme based on two cities’ administrative insurance claim datasets, and found that outpatient and inpatient care are substitutes for each other, and that reduced cost-sharing of outpatient care can help address its underutilization. Another study conducted similar research using the UEBMI claims database and came to the same conclusions (Zhu et al. 2021). Relatedly, Du *et al*. (2022) found a positive correlation between a decrease in cost-sharing for outpatient care for chronic disease-related services and a decreased hospitalization rate. However, these studies have limitations in that they explore simple associations and do not account for key socio-economic characteristics that could influence health service use, such as income, educational attainment of the patient and their household members, and household size.

Given the limitations, it is imperative to delve deeper into the effectiveness of cost-sharing within diverse socio-economic settings. This paper exploits the considerable variation that exists in cost-sharing across Chinese provinces/regions to examine the relationship between cost-sharing and the utilization of outpatient and inpatient health services. We ask whether there exists a substitution effect, where increased cost-sharing for outpatient care leads to increased inpatient service utilization (and vice versa), or a complementarity effect, wherein both service types are used jointly and an increase in either price reduces the utilization of both. We also contribute to the literature by taking account of the socio-economic characteristics of the population, providing insights into how different socio-economic groups are affected by cost-sharing changes. Our study further aims to identify separately the moral hazard effect and the reasonable utilization of healthcare demand, which is crucial for understanding the barriers to healthcare access and the potential overuse of services. The results of this study enable a greater understanding of how the detail of the cost-sharing rules of SHI schemes affects healthcare utilization in low- and middle-income countries.

## Conceptual framework

The standard theory of demand suggests that a reduction in the price of health services, such as outpatient consultations, will increase the demand for health services. The cross-price effect describes how the demand for health services responds to changes in the price of another good or service. Thus, the cost-sharing rate for a specific health service has the potential to impact not just the utilization of that service, but also services that are complements and/or substitutes for it.

Another relevant concept is that of moral hazard, which has been a key concern in health insurance design. When people are insured, they are more likely to use covered services, even if they are not entirely necessary, because the insured pay less for health care than it costs (Arrow 2004). Thus, higher cost-sharing for outpatient services may nudge individuals towards unnecessary inpatient service use, resulting in costs of healthcare that are higher than they would be otherwise, with associated implications for the financial sustainability of health insurance.

Given these theoretical foundations, we explore the following hypotheses in this paper.

(i) An increase in the cost-sharing level for outpatient services inversely impacts individuals’ utilization of these services.

(ii) Assuming the validity of the first point, an increase in outpatient services’ cost-sharing leads to a reduction in necessary inpatient service utilization, given that outpatient services often serve as prerequisites and/or a gateway for inpatient care.

(iii) If cost-sharing levels for outpatient services are high relative to those for inpatient services, individuals may be incentivized to over-utilize inpatient services.

## Materials and methods

### Data sources

The data used in this study were obtained from the 2015 China Health and Retirement Longitudinal Study (CHARLS). CHARLS is a nationwide survey focusing on the population aged ≥45 years in 150 counties from 28 provinces across China. The national baseline survey for CHARLS was conducted between June 2011 and March 2012, with subsequent follow-ups every 2 years. Currently, there are four waves of updated data available. CHARLS employs a stratified, multi-stage, and probability-proportionate-to-size sampling strategy. For detailed information on the CHARLS methodology, please refer to Zhao *et al*. 2014. The CHARLS survey is composed of several modules, each focusing on a specific area such as socio-economic status, healthcare utilization, health outcomes, health behaviours, familial support, and psychological well-being. Our study mainly relied on the data gathered on the ‘demographic background’, ‘health status and functioning’, and ‘health care and insurance’ modules of the 2015 CHARLS to analyse the association between cost-sharing and healthcare utilization of middle-aged and older residents. We opted for the data from the 2015 wave of the CHARLS datasets as it contains information about survey respondents who fell ill and did not seek outpatient care as well as information on those individuals who were advised by physicians to be hospitalized but did not receive inpatient treatment. These questions were changed in subsequent versions making it impossible to identify the role of physician advice in hospitalization.

Given our interest in the implications of cost-sharing, we restricted our attention to respondents who reported being covered by SHI. In addition, we excluded observations from Beijing and Shanghai, since only a single respondent in each location reported utilization of inpatient healthcare services and associated expenditures (94 observations). After removing responses that had missing values (53 observations), the final sample used for our analysis consisted of 15 362 individuals. We included data on the provision of healthcare resources in each of the surveyed provinces for the year 2015, as reported in the China Health Statistics Yearbook 2019, and we used the Consumer Price Index (CPI) of healthcare services for each province in 2015 obtained from the National Bureau of Statistics of China (National Bureau of Statistics of China 2021) to adjust for differences in the relative costs of healthcare across provinces.

### Variables

#### Outcome variables

Outcome variables included indicators of outpatient service utilization, unmet need for inpatient service (i.e. forgoing of doctor-initiated hospitalization), and any inpatient service utilization (including discretionary hospitalization and doctor-initiated hospitalization). Outpatient visits reported by the respondents in the 1 month preceding the survey were used as the indicator of outpatient service utilization. Hospitalization reported by the respondents in the last year preceding the survey was used as the indicator of inpatient service utilization.

#### Explanatory variables

The key explanatory variables of interest were a measure of the level of cost-sharing for outpatient care and a measure of the level of cost-sharing for inpatient care. The Chinese SHI system adopted a combination of deductible, coinsurance, and cap, with coverage differing based on the type of consultation, medications, and diagnostics for the outpatient and inpatient care services (Dong et al. 2023). For instance, in 2023, the URRBMI policy in Nanjing stipulated an outpatient deductible of RMB 200, with a maximum annual reimbursement limit of RMB 300 for the general population; for college students, the figures were RMB 100 and RMB 600, respectively; for individuals aged ≥80 years, the deductible and cap were set at RMB 200 and RMB 330, respectively (Nanjing Health Insurance 2023). Acknowledging the geographical variation in the cost-sharing levels in the Chinese system (Liu 2011), as well as the different details of the health insurance policies, we adopted the total actual reimbursement ratio as a proxy to gauge the level of patient cost-sharing effectively. Here cost-sharing is calculated to be the mean of the share of out-of-pocket expenditure in total expenditure across individuals for each province, weighted using individual sampling weights and adjusted for household and individual responses. Additionally, the ratio of cost-sharing for outpatient to inpatient care is utilized as a crucial explanatory variable to accurately depict the structural relationship between outpatient and inpatient cost-sharing rates.

While analysing CHARLS data on a city-by-city or province-by-province basis results in sample sizes too small for analysis, stratifying and classifying cost-sharing ratios into quartiles offers a viable solution. To capture variations in cost-sharing practices across different regions, we classified the province-level cost-sharing ratios into quartiles, separately for inpatient and outpatient services. Respondents were mapped to one of these quartiles, depending on their province of residence. The stratified indicators were used to assess the implications of cost-sharing practices for health service utilization at the individual level. This strategy of stratifying cost-sharing ratios into quartiles had the advantage of lowering sensitivity to outliers in our data, although it also reduced variation in cost-sharing measures and consequently the risk of noisiness of the key coefficients of interest.

#### Other explanatory variables

Standard methods in healthcare utilization research commonly identify predisposing factors, individual needs, and enabling resources as influential determinants in understanding healthcare utilization trends (Andersen 1995, Shao et al. 2018, Zeng et al. 2021). Predisposing factors include age, gender, education, and marital status, and have been shown to influence healthcare utilization in previous work. Individual needs factors included were general health status and the presence of chronic diseases. Enabling factors included were health insurance (type of SHI), health resources in the residential area, as measured by the number of physicians per 1000 population and the number of beds per 1000 population in each province, the number of dependent children, living conditions, family income [using total household per capita consumption (PCE) as a measure], and the availability of health resources in proximity. These variables were included as explanatory variables. To account for the regional disparities in healthcare service costs, we incorporated the health care CPI of the provinces into the model.

### Empirical analysis

We employed linear probability models in this study for their ease of interpretation and suitability for examining the relationship between the outcomes of interest and indicators of cost-sharing and other controls (Battey et al. 2019).

As explained in the conceptual framework, inpatient and outpatient services may be affected directly by their own price effects and by cross-price effects. Accordingly, we constructed different linear probability models for outpatient and inpatient care utilization respectively, which take the following forms.


(1) $${Y_{ihj}} = {\beta _0} + {\beta _1}{X_1}_{ihj} + {\beta _2}{X_2}_{ihj} + {\beta _3}{W_{ihj}} + {\varepsilon _{ihj}}$$

where, ${Y_{ihj}}$ refers to a binary outcome indicator of outpatient care utilization for a respondent *i* living in household *h* and province *j*. It takes the value 1 (utilized outpatient service) or 0 (did not utilize outpatient service). ${\beta _0}$ serves as the baseline probability of using outpatient services. ${X_1}_{ihj}$ is a measure of the cost-sharing level for out-patient care, and ${X_2}_{ihj}$ refers to the cost-sharing level for in-patient care. ${W_{ihj}}$ refers to socio-economic and demographic and other co-variates that could also influence the utilization of out-patient services. The model includes an error term, ${\varepsilon _{ihj}}$, which accounts for unobserved factors influencing health service utilization for individual *i* living in household *h* and province *j*.


(2) $${Z_{ihj\_1}} = {\beta _0} + {\beta _1}{X_2}_{ihj} + {\beta _2}{X_3}_{ihj} + {\beta _3}{W_{ihj}} + {\varepsilon _{ihj}}$$

According to the third hypothesis in this paper, we employed equation (2) to estimate the relationship between cost-sharing and inpatient service utilization in the case that a doctor’s recommendation for hospitalization is not followed. The structure of the equation is like that used for outpatient services, but with some key differences in terms of the explanatory variables. Here, ${Z_{ihj\_1}}$denotes a binary variable indicating whether individual *i* living in household *h* and province *j* utilized doctor-initiated inpatient services. Specifically, it takes the value 1 if doctor-initiated hospitalization is not taken up, or 0 for other scenarios. In this model, both the cost-sharing level for inpatient care ${X_2}_{ihj}$and the cross-price ratio, which is the ratio of outpatient cost-sharing to inpatient cost-sharing, ${X_3}_{ihj}$, are hypothesized to influence the utilization of inpatient care.


(3) $${Z_{ihj\_2}} = {\beta _0} + {\beta _1}{X_2}_{ihj} + {\beta _2}{X_3}_{ihj} + {\beta _3}{W_{ihj}} + {\varepsilon _{ihj}}$$

Extending from equation (2), we derive equation (3) by redefining the dependent variable, represented by ${Z_{ihj\_2}}$, to represent the total utilization of inpatient services, which includes both doctor-initiated and doctor non-initiated uses. Specifically, it takes the value 1 (utilized inpatient service) or 0 (did not utilize inpatient service). The broader definition of the dependent variable allows us to examine the effects of cost-sharing on overall inpatient service consumption, encompassing all situations where such services are utilized, irrespective of whether a doctor’s recommendation was present or not. All the independent variables here remain identical to those used in equation (2). This consistency allows for a direct comparison of the impact of these factors across different scopes of inpatient service utilization.

## Results

### Descriptive statistics

**Figure 2.**
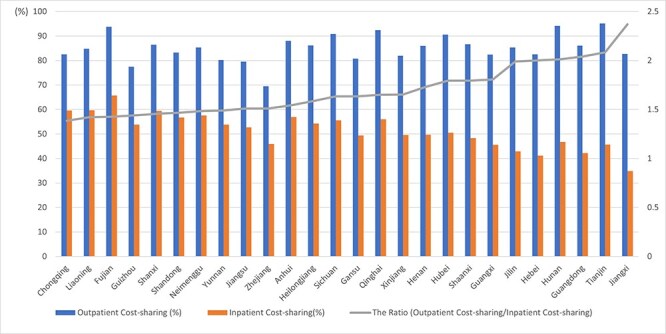
Cost-sharing by type of health service across provinces. Source: authors’ estimates using CHARLS data of 2015.

Figure 2 reports our findings on the levels of cost-sharing for different types of health services across 26 provinces. Interprovincial differences in cost-sharing levels for outpatient and inpatient care can be observed. For example, mean outpatient cost-sharing ranges from a low of 69.5 to a high of 95.2%. Inpatient cost-sharing is lower, and the means for inpatient cost-sharing range from 34.9 to 65.8%. Variations also exist across the ratios of the two sets of cost-sharing means—namely, the ratio between the cost-sharing levels of outpatient to inpatient services. Here the values vary from 1.38 to 2.37. The finding of higher cost-sharing for outpatient care relative to inpatient care is consistent with the priority given to financial protection for severe illnesses typically requiring inpatient care in the design of SHI in China.

Table 1 shows basic demographic and socio-economic characteristics for all 15 362 individuals in our final analytical sample, including by rural and urban residence. The average age of the sample respondents is ∼60 years. Gender distribution leans slightly towards women (52.2%), and a significant majority of respondents (86.5%) are married or partnered. When examining education levels, the sample reveals a relatively low level of formal education, with 60.0% of respondents having received an education in elementary school only or lower. This population is largely covered by the New Cooperative Medical Insurance (73.4%), which is mainly designed for rural residents. In terms of health status, a majority (78.0%) self-report their health as ‘fair and above’, with a chronic disease prevalence of 67.7%.

**Table 1.** Basic sample characteristics^a^

	Total (*n* = 15 362)	Urban (*n* = 5711)	Rural (*n* = 9651)
Age (mean, SD)	59.90 (0.16)	60.08 (0.26)	59.74 (0.20)
Gender			
Man	7381 (47.8%)	2678 (46.4%)	4703 (49.0%)
Woman	7981 (52.2%)	3033 (53.6%)	4948 (51.0%)
Marriage status			
Married/partnered	13 301 (86.5%)	4933 (86.4%)	8368 (86.7%)
Never married/ divorced/ separated	239 (1.6%)	101 (1.6%)	138 (1.6%)
Widowed	1822 (11.9%)	677 (12.0%)	1145 (11.8%)
Education			
Illiterate	3925 (22.8%)	946 (14.2%)	2979 (29.8%)
Can read and write	2797 (17.3%)	863 (13.3%)	1934 (20.5%)
Elementary school	3440 (22.0%)	1221 (20.4%)	2219 (23.4%)
Middle or high school	4567 (32.0%)	2187 (40.7%)	2380 (24.9%)
Vocational school or college and above	633 (5.9%)	494 (11.4%)	139 (1.4%)
Log PCE (mean, SD)	9.15 (0.03)	9.37 (0.04)	8.96 (0.02)
Self-reported health			
Fair and above	11 133 (78.0%)	4351 (82.9%)	6782 (74.0%)
Poor	3387 (22.0%)	988 (17.1%)	2399 (26.0%)
Noncommunicable disease			
No	4890 (32.3%)	1823 (32.5%)	3067 (32.2%)
Yes	10 472 (67.7%)	3888 (67.5%)	6584 (67.8%)
Number of living children			
0	141 (0.9%)	44 (0.5%)	97 (1.3%)
1	2179 (16.6%)	1321 (25.1%)	858 (9.7%)
2	5622 (36.6%)	2057 (36.5%)	3565 (36.7%)
3+	7420 (45.9%)	2289 (37.9%)	5131 (52.4%)
Social insurance type			
UEBMI	1982 (18.1%)	1697 (36.6%)	285 (3.2%)
URRBMI	1035 (8.5%)	832 (16.0%)	203 (2.4%)
NRCMI	12 345 (73.4%)	3182 (47.4%)	9163 (94.4%)

a Calculations were weighted using individual sampling weights and adjusted for household and individual responses.

### Association between cost-sharing level and outpatient service utilization

Table 2 presents the effects of varying levels of cost-sharing for both outpatient and inpatient services on the likelihood of individuals utilizing outpatient services when they fall ill. As the cost-sharing levels for outpatient services rise, the overall utilization of outpatient services tends to decrease. Conversely, an increase in the cost-sharing level for inpatient services corresponds with an increased likelihood of utilizing outpatient services, especially for rural residents.

**Table 2.** Association between cost-sharing level and outpatient services utilization^a^

	Total		Urban		Rural	
	Coef.	SE	Coef.	SE	Coef.	SE
Cost-sharing for outpatients						
Ref: Q1 (69.5–82.4%)						
Q2 (82.4–85.4%)	0.013	(0.034)	0.014	(0.065)	0.033	(0.039)
Q3 (85.4–88.0%)	−0.103**	(0.042)	−0.122*	(0.064)	−0.029	(0.046)
Q4 (88.0–95.2%)	−0.017	(0.041)	−0.083	(0.070)	−0.024	(0.033)
Cost-sharing for inpatients						
Ref: Q1(34.9–46.0%)						
Q2 (46.0–50.5%)	0.142***	(0.045)	0.112*	(0.065)	0.072	(0.048)
Q3 (50.5–56.8%)	0.126***	(0.038)	−0.065	(0.050)	0.153***	(0.046)
Q4 (56.8–65.8%)	0.100**	(0.048)	0.027	(0.067)	0.011	(0.046)
Age	0.002	(0.002)	0.002	(0.003)	0.003**	(0.002)
Marriage status						
Ref: married/partnered						
Never married/ divorced/separated	−0.096	(0.088)	−0.052	(0.152)	−0.088	(0.107)
Widowed	−0.065*	(0.040)	−0.047	(0.074)	−0.060	(0.040)
Gender						
Ref: male						
Female	0.020	(0.026)	0.006	(0.042)	0.026	(0.029)
Education						
Ref: illiterate						
Can read and write	0.013	(0.034)	0.126*	(0.071)	−0.012	(0.036)
Elementary school	0.017	(0.038)	0.080	(0.071)	0.008	(0.034)
Middle or high school	0.054	(0.036)	0.086	(0.072)	0.071**	(0.036)
Vocational school and above	0.153**	(0.064)	0.205**	(0.090)	−0.055	(0.099)
Number of living children						
Ref: 0						
1	0.153	(0.114)	−0.006	(0.165)	0.087	(0.130)
2	0.107	(0.110)	−0.064	(0.161)	0.118	(0.124)
≥3	0.091	(0.112)	−0.180	(0.164)	0.148	(0.127)
Log PCE	0.008	(0.013)	0.009	(0.020)	0.014	(0.013)
Self-reported health						
Ref: fair and above						
Poor	−0.017	(0.022)	−0.025	(0.041)	−0.027	(0.023)
Noncommunicable disease						
Ref: no						
Yes	0.069**	(0.029)	0.111**	(0.050)	0.047	(0.030)
Health resource						
Physicians per 1000, city	0.030	(0.022)	−0.016	(0.032)		
Beds per 1000, city	−0.017*	(0.010)	−0.028	(0.018)		
Physicians per 1000, rural	0.125***	(0.035)			0.084*	(0.044)
Beds per 1000, rural	−0.136***	(0.036)			−0.125***	(0.036)
Social insurance type						
Ref: UEBMI						
URRBMI	0.055	(0.089)	0.036	(0.098)	0.127	(0.100)
NRCMI	0.121*	(0.064)	0.160**	(0.077)	0.043	(0.071)
Living area						
Ref: urban						
Rural	−0.006	(0.029)				
Constant	1.267	(1.528)	−0.574	(2.552)	−0.129	(1.415)
Observations	2938		1043		1895	
*R* ^2^	0.031		0.077		0.032	

*** *P* < 0.01,

** *P* < 0.05,

* *P* < 0.1.

a Robust standard errors (SEs) (clustered at the community level) are in parentheses.

Estimates were weighted using individual sampling weights and adjusted for household and individual responses.

The results have been adjusted for the CPI of healthcare service for each province.

The availability of physicians and beds in rural areas also has a significant impact. For every additional physician per 1000 people in a rural area, the likelihood of utilizing outpatient services increases 12.5%. Conversely, for every additional bed per 1000 people in a rural area, the likelihood of utilizing outpatient services decreases 13.6% significantly. The health resources by city area show the same trends, but these results are not statistically significant. Other factors such as higher education level and having a noncommunicable disease are also associated with higher probability of use of outpatient services.

### Association between cost-sharing level and forgone doctor-initiated hospitalization

Table 3 presents the association between inpatient cost-sharing level, the ratio of outpatient cost-sharing to inpatient cost-sharing, and the forgoing of a doctor-initiated hospitalization. The data indicate an increased likelihood of forgone doctor-initiated hospitalization as the inpatient cost-sharing rate rises, with this trend being significant for the total population and rural residents at the Q3 (50.5–56.8%) and Q4 (56.8–65.8%) levels.

**Table 3.** Association between cost-sharing level and unmet inpatient services^a^

	Total		Urban		Rural	
	Coef.	SE	Coef.	SE	Coef.	SE
Cost-sharing for inpatients						
Ref: Q1(34.9–46.0%)						
Q2 (46.0–50.5%)	0.006	(0.012)	0.001	(0.022)	0.017	(0.013)
Q3 (50.5–56.8%)	0.038***	(0.013)	−0.000	(0.015)	.052***	(0.019)
Q4 (56.8–65.8%)	0.045***	(0.016)	−0.004	(0.019)	0.076***	(0.022)
Outpatient/inpatient cost-sharing ratio						
Ref: Q1 (1.38–1.48)						
Q2 (1.48–1.64)	0.015	(0.009)	−0.003	(0.012)	0.030**	(0.012)
Q3 (1.64–1.80)	0.065***	(0.016)	0.025	(0.028)	0.062**	(0.024)
Q4 (1.80–2.37)	0.062***	(0.015)	0.005	(0.018)	0.069***	(0.021)
Age	−0.000	(0.000)	−0.000	(0.001)	−0.000	(0.000)
Marriage status						
Ref: married/partnered						
Never married/ divorced/separated	−0.001	(0.025)	0.003	(0.040)	−0.000	(0.031)
Widowed	−0.010	(0.010)	−0.015	(0.014)	−0.007	(0.014)
Gender						
Ref: male						
Female	0.007	(0.006)	0.007	(0.009)	0.009	(0.008)
Education						
Ref: illiterate						
Can read and write	−0.000	(0.008)	−0.013	(0.015)	0.007	(0.009)
Elementary school	−0.010	(0.008)	−0.014	(0.014)	−0.009	(0.010)
Middle or high school	−0.006	(0.009)	−0.009	(0.017)	−0.005	(0.011)
Vocational school and above	0.015	(0.015)	0.021	(0.021)	−0.027	(0.019)
Number of living children						
Ref: 0						
1	0.007	(0.034)	−0.075	(0.108)	0.046	(0.029)
2	0.003	(0.033)	−0.074	(0.107)	0.031	(0.029)
≥3	0.008	(0.034)	−0.068	(0.109)	0.033	(0.029)
Log PCE	0.002	(0.002)	−0.002	(0.004)	0.003	(0.003)
Self-reported health						
Ref: fair and above						
Poor	0.085***	(0.009)	0.076***	(0.017)	0.091***	(0.010)
Noncommunicable disease						
Ref: no						
Yes	0.036***	(0.005)	0.036***	(0.006)	0.036***	(0.006)
Health resource						
Physicians per 1000, city	−0.013***	(0.004)	−0.010*	(0.005)		
Beds per 1000, city	−0.000	(0.002)	0.009**	(0.004)		
Physicians per 1000, rural	0.020**	(0.010)			0.019	(0.016)
Beds per 1000, rural	0.024***	(0.007)			0.017*	(0.010)
Social insurance type						
Ref: UEBMI						
URRBMI	0.025**	(0.012)	0.034**	(0.014)	−0.010	(0.028)
NRCMI	0.019**	(0.009)	0.028**	(0.012)	−0.003	(0.016)
Living area						
Ref: urban						
Rural	0.005	(0.006)				
Constant	−1.303***	(0.303)	−0.444	(0.503)	−1.072***	(0.368)
Observations	10 026		3761		6265	
*R* ^2^	0.044		0.043		0.045	

*** *P* < 0.01,

** *P* < 0.05,

* *P* < 0.1.

a Robust standard errors (SEs) (clustered at the community level) are in parentheses.

Estimates were weighted using individual sampling weights and adjusted for household and individual responses.

The results have been adjusted for the CPI of healthcare service for each province.

In addition, as the ratio of outpatient cost-sharing to inpatient cost-sharing rises, there is an observed increase in the likelihood of unmet hospitalizations. This trend is notably evident starting from the Q3 level for the entire population, where the average outpatient cost-sharing level is 1.64 to 1.80 times that of the average inpatient cost-sharing level. At the Q3 and Q4 ratio levels, the result for the total population indicates an ∼6.5 and 6.2% rise, respectively, in forgone doctor-initiated hospitalization when compared to the baseline group Q1. Similarly, for rural residents, there is a corresponding 6.2 and 6.9% increase in forgone doctor-initiated hospitalization at the Q3 and Q4 levels, respectively, compared to the reference group.

A positive relationship is observed between healthcare resources, specifically the number of beds per thousand population in city areas, and forgone doctor-initiated hospitalization. This may suggest inefficiencies in healthcare resource utilization in urban settings. Regarding other variables, households with URRBMI or NRCMI have a higher likelihood of forgoing doctor-initiated hospitalization compared to UEBMI. Additionally, a poor self-reported health status and the presence of noncommunicable diseases are positively associated with this likelihood.

### Association between cost-sharing level and overall inpatient services utilization

To understand the relationship between cost-sharing level and overall inpatient service utilization, we utilized the same set of variables as those examined in Table 3. The results show that there is no significant relationship between cost-sharing levels for inpatients and the utilization of inpatient services overall. However, a notable positive association was observed for the ratio of outpatient to inpatient cost-sharing and the overall inpatient service utilization. In particular, when the outpatient to inpatient cost-sharing ratio is high (Q3, ranging from 1.64 to 1.80 times), the probability of using inpatient services rises by 7.7% compared to the reference group (Q1, ranging from 1.38 to 1.48 times). For rural residents, this likelihood further increases by 6.5 and 5.3% respectively when the ratio reaches the Q3 and Q4 levels.

As for other control variables, the signs of coefficients are as expected. Specifically, the likelihood of hospitalization utilization was higher for older people, those with higher income, poor self-reported health, and presence of noncommunicable diseases. Additionally, living in areas with a greater number of beds per thousand population was also positively related to higher overall hospitalization rates (Table 4).

**Table 4.** Association between cost-sharing level and overall inpatient services utilization^a^

	Total		Urban		Rural	
	Coef.	SE	Coef.	SE	Coef.	SE
Cost-sharing for inpatients						
Ref: Q1(34.9–46.0%)						
Q2 (46.0–50.5%)	−0.036	(0.020)	−0.028	(0.026)	−0.006	(0.022)
Q3 (50.5–56.8%)	0.017	(0.025)	0.044	(0.034)	0.023	(0.023)
Q4 (56.8–65.8%)	−0.010	(0.030)	−0.002	(0.041)	0.017	(0.026)
Outpatient/inpatient cost-sharing ratio						
Ref: Q1 (1.38–1.48)						
Q2 (1.48–1.64)	0.005	(0.015)	0.001	(0.025)	0.019	(0.016)
Q3 (1.64–1.80)	0.077***	(0.027)	0.086**	(0.043)	0.065**	(0.030)
Q4 (1.80–2.37)	0.017	(0.025)	−0.003	(0.041)	0.053**	(0.026)
Age	0.004***	(0.001)	0.004***	(0.001)	0.003***	(0.001)
Marriage status						
Ref: married/partnered						
Never married/ divorced/separated	0.021	(0.040)	−0.029	(0.061)	0.079	(0.054)
Widowed	0.004	(0.017)	−0.026	(0.030)	0.031*	(0.018)
Gender						
Ref: male						
Female	0.015*	(0.008)	0.017	(0.014)	0.014	(0.010)
Education						
Ref: illiterate						
Can read and write	−0.010	(0.013)	−0.026	(0.027)	−0.007	(0.014)
Elementary school	0.014	(0.012)	0.005	(0.026)	0.014	(0.013)
Middle or high school	0.006	(0.012)	−0.012	(0.022)	0.009	(0.013)
Vocational school and above	−0.041**	(0.020)	−0.065**	(0.027)	0.017	(0.040)
Number of living children						
Ref: 0						
1	0.005	(0.053)	−0.038	(0.113)	0.067	(0.066)
2	0.015	(0.052)	−0.014	(0.113)	0.05	(0.064)
≥3	0.021	(0.053)	−0.032	(0.116)	0.074	(0.065)
Log PCE	0.035***	(0.006)	0.030***	(0.011)	0.038***	(0.005)
Self-reported health						
Ref: fair and above						
Poor	0.158***	(0.012)	0.183***	(0.021)	0.145***	(0.014)
Noncommunicable disease						
Ref: no						
Yes	0.052***	(0.008)	0.052***	(0.014)	0.052***	(0.009)
Health resource						
Physicians per 1000, city	−0.026***	(0.009)	−0.029**	(0.012)		
Beds per 1000, city	0.009**	(0.004)	0.007	(0.007)		
Physicians per 1000, rural	−0.025	(0.017)			−0.038**	(0.019)
Beds per 1000, rural	0.034**	(0.015)			0.037**	(0.018)
Social insurance type						
Ref: UEBMI						
URRBMI	0.005	(0.018)	0.004	(0.020)	0.043	(0.040)
NRCMI	−0.038**	(0.017)	−0.045**	(0.022)	−0.018	(0.025)
Living area						
Ref: urban						
Rural	−0.002	(0.009)				
Constant	−1.062**	(0.524)	0.131	(1.009)	−0.991*	(0.529)
Observations	10 027		3761		6266	
*R* **^2^**	0.083		0.093		0.081	

*** *P* < 0.01,

** *P* < 0.05,

* *P* < 0.1.

a Robust standard errors (SEs) (clustered at the community level) are in parentheses.

Estimates were weighted using individual sampling weights and adjusted for household and individual responses.

The results have been adjusted for the CPI of healthcare service for each province.

### Healthcare utilization among patients with chronic disease

Given the long-term treatment required for chronic diseases, patients with such conditions may be more price sensitive. We hypothesise that when the cost-sharing for outpatient services significantly exceeds that for inpatient services, patients with chronic diseases have an elevated tendency to favour inpatient over outpatient services. We focus on the patients with hypertension and/or diabetes, the two most prevalent chronic conditions in China, to check whether the health service utilization of this group aligns with this hypothesis.

Table 5 shows the association between the cost-sharing level and outpatient service utilization among patients with hypertension and/or diabetes. As the level of cost-sharing for outpatients increases, patients with hypertension and/or diabetes have a lower probability of using outpatient services. Conversely, as the level of cost-sharing for inpatients increases, patients with hypertension and/or diabetes have a higher probability of using outpatient services. However, this pattern is not significant for other individuals.

**Table 5.** Association between cost-sharing level and outpatient services utilization among patients with chronic diseases (hypertension and/or diabetes)^a^

	Chronic patients		Others	
	Coef.	SE	Coef.	SE
Cost-sharing for outpatients				
Ref: Q1 (69.5–82.4%)				
Q2 (82.4–85.4%)	0.029	(0.047)	0.019	(0.046)
Q3 (85.4–88.0%)	−0.136**	(0.060)	−0.048	(0.051)
Q4 (88.0–95.2%)	−0.035	(0.058)	0.034	(0.050)
Cost-sharing for inpatients				
Ref: Q1(34.9–46.0%)				
Q2 (46.0–50.5%)	0.191***	(0.065)	0.067	(0.056)
Q3 (50.5–56.8%)	0.156***	(0.052)	0.104*	(0.055)
Q4 (56.8–65.8%)	0.147**	(0.059)	0.048	(0.069)
Control variables	Yes		Yes	
Constant	1.868	(2.260)	1.287	(2.552)
Observations	1206		1593	
*R* ^2^	0.048		0.030	

*** *P* < 0.01,

** *P* < 0.05,

* *P* < 0.1.

a Robust standard errors (SEs) (clustered at the community level) are in parentheses.

Estimates were weighted using individual sampling weights and adjusted for household and individual responses.

The results have been adjusted for the CPI of healthcare service for each province.

The results in Table 6 show that both individuals with chronic diseases and other individuals are influenced by the structure of cost-sharing, with chronic disease patients particularly sensitive to increases in both inpatient cost-sharing levels and the ratio of outpatient to inpatient cost-sharing, resulting in a higher likelihood of forgoing inpatient services. For chronic patients, as the inpatient cost-sharing level increases from Q1 to Q3 and Q4, there is a corresponding 6.1 and 7.8% increase in forgone doctor-initiated hospitalization, respectively, compared to the reference group. A similar, albeit less pronounced, pattern is observed for other individuals, with a 3.2% significant increase in forgoing doctor-initiated hospitalization at the Q3 level compared to the reference group.

**Table 6.** Association between cost-sharing level and inpatient services utilization among patients with chronic diseases (hypertension or diabetes)^a^

	Chronic patients		Others	
	Forgone doctor- initiated hospitalization (Coef. /SE)	Overall Hospitalization (Coef. /SE)	Forgone doctor- initiated hospitalization (Coef. /SE)	Overall Hospitalization (Coef. /SE)
Cost-sharing for inpatients				
Ref: Q1(34.9–46.0%)				
Q2 (46.0–50.5%)	0.041 (0.027)	−0.067* (0.039)	−0.005 (0.012)	−0.027 (0.018)
Q3 (50.5–56.8%)	0.061*** (0.023)	0.033 (0.046)	0.032** (0.014)	−0.012 (0.026)
Q4 (56.8–65.8%)	0.078*** (0.029)	0.009 (0.055)	0.033* (0.019)	−0.039 (0.031)
Outpatient/inpatient cost-sharing ratio				
Ref: Q1 (1.38–1.48)				
Q2 (1.48–1.64)	0.020 (0.017)	0.007 (0.029)	0.011(0.011)	0.009 (0.016)
Q3 (1.64–1.80)	0.075**(0.033)	0.170*** (0.052)	0.062*** (0.018)	0.024 (0.030)
Q4 (1.80–2.37)	0.099***(0.027)	0.077 (0.047)	0.051*** (0.017)	−0.015 (0.029)
Control variables	Yes	Yes	Yes	Yes
Constant	−0.508(0.608)	−0.891 (1.239)	−0.561* (0.334)	−1.506*** (0.563)
Observations	3583	3583	5956	5957
*R* ^2^	0.042	0.071	0.042	0.076

*** *P* < 0.01,

** *P* < 0.05,

* *P* < 0.1.

a Robust standard errors (SEs) (clustered at the community level) are in parentheses.

Estimates were weighted using individual sampling weights and adjusted for household and individual responses.

The results have been adjusted for the CPI of healthcare service for each province.

For overall hospitalization, the result is similar to the whole population, in that there is no significant relationship between cost-sharing levels for inpatients and the utilization of overall inpatient services. However, for chronic patients, a positive association was observed for the ratio of outpatient to inpatient cost-sharing and overall inpatient service utilization, while a positive trend is not found for others. These results are consistent with our hypothesis and provide further evidence for the substitution effect between outpatient and inpatient service utilization.

## Discussion and conclusion

In this study, we investigated the relationship between cost-sharing and the utilization of outpatient and inpatient services in China, drawing upon the frameworks of the standard theory of demand and the theory of moral hazard. Our findings reveal a complex relationship that supports the hypothesis that both substitution and complementarity effects exist in the Chinese healthcare context.

We identified a substitution relationship between outpatient and inpatient services in two key respects. First, we observed that elevated levels of cost-sharing for outpatient services were associated with an increase in the overall utilization of inpatient care, while the level of inpatient cost-sharing did not exhibit a significant correlation with overall inpatient service utilization. This substitution effect is consistent with some studies in other countries (Fusco et al. 2023) and several studies in China which only focused on one specific SHI type (Zhu et al. 2021, He 2022). This finding reinforces the idea that patients might substitute more costly and potentially unnecessary inpatient services for outpatient care when faced with higher cost-sharing for outpatient services.

This result aligns with another finding from our study, a substantial positive correlation between the number of beds per thousand population in the respondent’s living region and the overall utilization rate of inpatient services. However, this correlation does not extend to doctor-initiated hospitalization, suggesting that an increase in inpatient resources does not effectively address residents’ hospitalization barriers. One interpretation is that the availability of inpatient resources from the supply side might pave the way for the provision of unnecessary inpatient services while failing to support access to services for those more likely to have a clinical need for hospitalization. Other interpretations are possible given understandings of levels of supplier-induced demand in China (Yu et al. 2020, Shen et al. 2024), but we consider it is likely that doctor-initiated hospitalizations are more clinically necessary than discretionary hospitalizations.

Second, our findings tentatively enrich the existing literature suggesting a potential trend in China, especially among rural residents: an increase in the likelihood of patients utilizing outpatient services as the cost-sharing burden for inpatient services rises. This suggests that rural patients are more susceptible to the impacts of rising costs for inpatient care. Faced with escalating financial barriers, rural residents might be more inclined to choose less costly outpatient alternatives, potentially compromising clinical outcomes in the process.

Simultaneously, our study introduced a novel observation that the likelihood of forgone doctor-initiated hospitalization is positively correlated with the ratio of outpatient to inpatient cost-sharing. This suggests the presence of complementary effects between the two types of services. Our unique finding adds a new dimension to the current body of knowledge which often treats inpatient service utilization as an aggregate variable without distinguishing between doctor-initiated and discretionary forms of inpatient care. When considered alongside the pattern in relation to overall inpatient service utilization, our findings suggest that as the cost-sharing of outpatient care increases, the observed increases in inpatient care utilization might reflect an escalation in moral hazard instead of a correction of the under-use of inpatient care. It seems possible that this phenomenon exists in China due to the significant disparities in outpatient and inpatient service cost sharing inherent in the Chinese SHI reimbursement policies. In the long-term, if the financial protection for outpatient services is low, due to individuals ignoring minor illnesses, it could contribute to an escalation in major health conditions and exacerbate the burden on hospital admissions (Gruber et al. 2020). Our study also found that patients with chronic diseases are more sensitive to the level of cost-sharing.

Our study also found high-income individuals have a higher probability of using inpatient services, consistent with findings from Kato and Goto (2017) that low-income groups tend be more responsive to charges due to their budget constraints. Our study also found urban–rural differences in the likelihood of hospitalization, with rural residents having a higher probability of needing but not receiving inpatient care compared to their urban counterparts. This aligns with (Zhang, 2020), who found that middle-aged and elderly populations in rural China, with typically lower income levels and health literacy, tend to under-utilize healthcare services until serious health issues arise. The findings highlight the need to improve equitable access to inpatient care in rural areas.

These findings have major policy implications. In the first place, they suggest that the emphasis placed on financial protection for individuals with more severe illness requiring hospitalization is yielding paradoxical results. Its effect seems to be to reduce hospitalization for those more likely to have a clinical need for hospitalization and increase it for those choosing to substitute inpatient for outpatient care in the absence of that clinical need, and the effect is pronounced in relation to excluding rural health system users and those with chronic illness from inpatient care. This is a significant issue for the equity of the system. In the second place, the outcome is unsustainable and inefficient. Substituting more costly and less efficient inpatient care for less costly and more efficient outpatient care is clearly unwise and reduces the Chinese system’s ability to achieve universal health coverage across all three dimensions of the universal health coverage ‘cube’ (Tangcharoensathien et al. 2016). Both problems should prompt Chinese health policy makers at city, provincial, and national levels to reconsider low reimbursement rates for outpatient care.

Our study offers national-level evidence indicating that increased cost-sharing deters the utilization of health services, and it identifies critical issues existing within the Chinese healthcare system that demand targeted policy interventions. The study emphasizes the need for comprehensive insurance coverage, with a greater level of effective coverage for high-value outpatient services, and suggests this as an effective strategy to mitigate such substitution effects.

While the data utilized in this study originates from 2015, it is essential to acknowledge some subsequent changes to SHI arrangements in China. These include the implementation of off-site medical settlement health insurance policies across some cities and an increase in the level of health insurance financing and coverage for outpatient services (Liu et al. 2017). Nevertheless, these reforms are still in the process of being progressively implemented, with differentiated application across various regions, and the cost-sharing mechanisms between outpatient and inpatient service for patients have not changed significantly. Thus, the structural challenges and patient behaviours identified in the 2015 data remain pertinent.

To our knowledge, this is the first study that delivers an in-depth analysis of the impact of Chinese SHI cost-sharing on health service utilization based on nationwide data, taking into account coverage variation across insurance types and geographical areas as well as individual socio-economic factors. Further, our methodology refines the definition of the dependent variable by focusing on forgone doctor-initiated hospitalization which may identify unmet need for inpatient services. This approach offers a more direct examination of potential moral hazard effects.

The results from this paper should be interpreted with caution due to certain limitations. First, the research might be affected by measurement errors on cost-sharing due to reliance on self-reported survey data, although our use of province-level indicators and quartiles of cost-sharing may have ameliorated that somewhat. Second, our sample population is primarily made up of elderly individuals, so the results may not be generalizable to the entire population. Our use of cross-sectional (as against longitudinal) data was driven by the availability of the key outcome variables. Aside from the 2015 dataset, other waves do not contain information about respondents who fell ill but did not access outpatient care, as well as details on individuals who forwent doctor-initiated hospitalization. This limited our ability to address omitted variable bias arising from factors such as severity of health conditions or individual-specific differences simultaneously driving hospital use and healthcare expenditures. In these circumstances, an instrumental variable (IV) approach is often the preferred option, but no obvious IV could be identified from the CHARLS data. Moreover, as Young (2022) shows, as a practical matter, IV estimates can be sensitive to outlying observations, and in most practical situations not statistically distinguishable from ordinary least squares estimates such as we report here. These limitations highlight areas for future research to address and further our understanding of the implications of cost-sharing policies in the Chinese healthcare system.

Although this study focuses on the Chinese context, the findings highlight the importance of balancing cost-sharing with healthcare services access, particularly for low-socio-economic populations, and addressing the moral hazard effect that leads to unnecessary service utilization. These insights are equally relevant for countries transitioning towards universal health coverage or seeking to optimize their existing health insurance systems

## Supplementary Material

czae108_Supp

## Data Availability

The data underlying this article are available on the China Health and Retirement Longitudinal Study website, at https://charls.charlsdata.com/pages/data/111/en.html.

## Reflexivity statement

The authors include two females and two males, with professional experience spanning multiple levels. While all authors specialize in various areas of health policy, health economics, and health systems, two of the authors have a specific focus on China’s health policy and have extensive experience of conducting fieldwork in China. The other two possess deep expertise in health economics and global health in Asia and Africa.

## Abbreviations

CHARLS: China Health and Retirement Longitudinal Study; SHI: Social Health Insurance; NRCMI: New Rural Cooperative Medical Insurance; UEBMI: Urban Employee Basic Medical Insurance; URRBMI: Urban and Rural Resident Basic Medical Insurance; CPI: consumer price index; PCE: per capita consumption; IV: instrumental variable; OECD: Organization for Economic Cooperation and Development.
