# Supplementary material for: A Chinese conundrum: does higher insurance coverage for hospitalization reduce financial protection for the patients who most need it?
Source: Health Policy Plan. 2024 Nov 9;40(3):287–99. doi: 10.1093/heapol/czae108 (PMC11886816; doi:10.1093/heapol/czae108)
Supplement: czae108_Supp [file czae108_supp.zip › New folder/Supplementary files.docx]

**Supplementary files**

S1. Flowchart of Sample Selection

94 from Beijing or Shanghai were excluded due to each having only one valid sample

All observations

N=21,098

Observations left

N=15,509

3,977 were excluded due to missing data on insurance participation;

1,612 were excluded due to not being covered by any social health insurance scheme

Sample size N=15,362

Observations left

N=15,415

53 were excluded due to missing values

S2. Association between Cost-Sharing Level and Outpatient Services Utilization among Patients with Chronic Diseases (with hypertension and/or diabetes)

|  |  | Chronic patients | |  | Others | | |
| --- | --- | --- | --- | --- | --- | --- | --- |
|  |  | Coef. SE | |  | Coef. SE | | |
| **Cost-sharing for outpatients** |  |  |  |  |  | |  |
| Ref: Q1 (69.5%-82.4%) |  |  |  |  |  | |  |
| Q2 (82.4%-85.4%) |  | 0.029 | (0.047) |  | | 0.019 | (0.046) |
| Q3 (85.4%-88.0%) |  | -0.136** | (0.060) |  | -0.048 | | (0.051) |
| Q4 (88.0%-95.2%) |  | -0.035 | (0.058) |  | 0.034 | | (0.050) |
| **Cost-sharing for inpatients** |  |  |  |  |  | |  |
| Ref: Q1(34.9%-46.0%) |  |  |  |  |  | |  |
| Q2 (46.0%-50.5%) |  | 0.191*** | (0.065) |  | 0.067 | | (0.056) |
| Q3 (50.5%-56.8%) |  | 0.156*** | (0.052) |  | 0.104* | | (0.055) |
| Q4 (56.8%-65.8%) |  | 0.147** | (0.059) |  | 0.048 | | (0.069) |
| **Age** |  | 0.000 | (0.002) |  | 0.001 | | (0.002) |
| **Marriage Status** |  |  |  |  |  | |  |
| Ref: Married/partnered |  |  |  |  |  | |  |
| Never married/ divorced/separated |  | -0.127 | (0.144) |  | 0.054 | | (0.127) |
| Widowed |  | -0.062 | (0.053) |  | -0.069 | | (0.055) |
| **Gender** |  |  |  |  |  | |  |
| Ref: Male |  |  |  |  |  | |  |
| Female |  | 0.022 | (0.039) |  | 0.033 | | (0.030) |
| **Education** |  |  |  |  |  | |  |
| Ref: Illiterate |  |  |  |  |  | |  |
| Can read and write |  | -0.009 | (0.050) |  | 0.037 | | (0.048) |
| Elementary school |  | -0.010 | (0.043) |  | 0.030 | | (0.056) |
| Middle or high school |  | -0.016 | (0.054) |  | 0.107** | | (0.052) |
| Vocational school and above |  | 0.092 | (0.071) |  | 0.163 | | (0.105) |
| **Number of living children** |  |  |  |  |  | |  |
| Ref: 0 |  |  |  |  |  | |  |
| 1 |  | -0.027 | (0.179) |  | 0.362* | | (0.204) |
| 2 |  | -0.047 | (0.174) |  | 0.324 | | (0.199) |
| ≥3 |  | -0.096 | (0.175) |  | 0.331* | | (0.199) |
| **Log (PCE)** |  | -0.010 | (0.022) |  | 0.009 | | (0.015) |
| **Self-reported health** |  |  |  |  |  | |  |
| Ref: Fair and above |  |  |  |  |  | |  |
| Poor |  | -0.062* | (0.036) |  | 0.043 | | (0.031) |
| **Health resource** |  |  |  |  |  | |  |
| Phys per 1000_City |  | 0.034 | (0.029) |  | 0.042 | | (0.032) |
| Beds per 1000_City |  | -0.016 | (0.013) |  | -0.027* | | (0.015) |
| Phys per 1000_Rural |  | 0.080* | (0.046) |  | 0.127** | | (0.054) |
| Beds per 1000_ Rural |  | -0.140*** | (0.052) |  | -0.135*** | | (0.048) |
| **Social insurance type** |  |  |  |  |  | |  |
| Ref: UEBMI |  |  |  |  |  | |  |
| URRBMI |  | -0.064 | (0.086) |  | 0.093 | | (0.106) |
| NRCMI |  | 0.082 | (0.063) |  | 0.104 | | (0.075) |
| **Living area** |  |  |  |  |  | |  |
| Ref: Urban |  |  |  |  |  | |  |
| Rural |  | -0.037 | (0.033) |  | 0.020 | | (0.043) |
| **Constant** |  | 1.868 | (2.260) |  | 1.287 | | (2.552) |
| **Observations** |  | 1206 |  |  | 1593 | |  |
| **R-squared** |  | 0.048 |  |  | 0.030 | |  |

*** p<0.01, ** p<0.05, * p<0.1

Robust SEs (clustered at the community level) are in parentheses.

Estimates were weighted using individual sampling weights and adjusted for household and individual responses.

The results presented in this table have been adjusted for the CPI of healthcare service for each province.

S3. Association between Cost-Sharing Level and Inpatient Services Utilization among Patients with Chronic Diseases (with hypertension or diabetes)

|  | Chronic patients | | Others | |
| --- | --- | --- | --- | --- |
|  | Forgone doctor- initiated hospitalisation (Coef. /SE) | Overall Hospitalisation (Coef. /SE) | Forgone doctor- initiated hospitalisation (Coef. /SE) | Overall Hospitalisation (Coef. /SE) |
| **Cost-sharing for inpatients** |  |  |  |  |
| Ref: Q1(34.9%-46.0%) |  |  |  |  |
| Q2 (46.0%-50.5%) | 0.041(0.027) | -0.067*(0.039) | -0.005(0.012) | -0.027(0.018) |
| Q3 (50.5%-56.8%) | 0.061***(0.023) | 0.033(0.046) | 0.032**(0.014) | -0.012(0.026) |
| Q4 (56.8%-65.8%) | 0.078***(0.029) | 0.009(0.055) | 0.033*(0.019) | -0.039(0.031) |
| **Outpatient/Inpatient Cost-sharing Ratio** |  |  |  |  |
| Ref: Q1 (1.38-1.48) |  |  |  |  |
| Q2 (1.48-1.64) | 0.020 (0.017) | 0.007(0.029) | 0.011(0.011) | 0.009(0.016) |
| Q3 (1.64-1.80) | 0.075**(0.033) | 0.170***(0.052) | 0.062***(0.018) | 0.024(0.030) |
| Q4 (1.80-2.37) | 0.099***(0.027) | 0.077(0.047) | 0.051***(0.017) | -0.015(0.029) |
| **Age** | -0.000(0.001) | 0.004***(0.001) | 0.000(0.000) | 0.003***(0.001) |
| **Marriage Status** |  |  |  |  |
| Ref: Married/partnered |  |  |  |  |
| Never married/ divorced/separated | -0.009(0.051) | -0.016(0.066) | 0.007(0.024) | 0.075(0.049) |
| Widowed | -0.019(0.016) | -0.010(0.030) | -0.008(0.011) | 0.018(0.020) |
| **Gender** |  |  |  |  |
| Ref: Male |  |  |  |  |
| Female | 0.010(0.011) | 0.003(0.018) | 0.006(0.006) | 0.022**(0.009) |
| **Education** |  |  |  |  |
| Ref: Illiterate |  |  |  |  |
| Can read and write | 0.003(0.015) | -0.010(0.027) | -0.001(0.009) | -0.012(0.014) |
| Elementary school | -0.007(0.015) | -0.002(0.024) | -0.006(0.009) | 0.021(0.013) |
| Middle or high school | -0.003(0.017) | 0.003(0.025) | -0.007(0.010) | 0.008(0.013) |
| Vocational school and above | 0.044(0.027) | -0.041(0.045) | 0.006(0.016) | -0.033(0.023) |
| **Number of living children** |  |  |  |  |
| Ref: 0 |  |  |  |  |
| 1 | 0.075**(0.034) | -0.051(0.096) | -0.044(0.054) | 0.045(0.075) |
| 2 | 0.066**(0.031) | -0.026(0.094) | -0.047(0.053) | 0.042(0.074) |
| ≥3 | 0.057*(0.033) | -0.033(0.095) | -0.033(0.054) | 0.054(0.074) |
| **Log (PCE)** | -0.004(0.004) | 0.037***(0.010) | 0.006**(0.003) | 0.036***(0.005) |
| **Self-reported health** |  |  |  |  |
| Ref: Fair and above |  |  |  |  |
| Poor | 0.092***(0.014) | 0.154***(0.021) | 0.092***(0.011) | 0.163***(0.016) |
| **Health resource** |  |  |  |  |
| Phys per 1000_City | -0.010(0.007) | -0.055***(0.016) | -0.013**(0.005) | -0.009(0.009) |
| Beds per 1000_City | -0.004(0.004) | 0.018**(0.008) | 0.002(0.003) | 0.003(0.005) |
| Phys per 1000_Rural | 0.040**(0.017) | -0.030(0.031) | 0.009(0.013) | -0.035*(0.018) |
| Beds per 1000_ Rural | 0.030**(0.013) | 0.047(0.030) | 0.024***(0.009) | 0.041***(0.013) |
| **Social insurance type** |  |  |  |  |
| Ref: UEBMI |  |  |  |  |
| URRBMI | 0.025(0.019) | 0.002(0.036) | 0.027*(0.014) | 0.005(0.020) |
| NRCMI | 0.021(0.017) | -0.056(0.035) | 0.017*(0.009) | -0.013(0.017) |
| **Living area** |  |  |  |  |
| Ref: Urban |  |  |  |  |
| Rural | 0.019(0.012) | 0.001(0.017) | 0.003(0.006) | -0.001(0.010) |
| **Constant** | -0.508(0.608) | -0.891(1.239) | -0.561*(0.334) | -1.506***(0.563) |
| **Observations** | 3583 | 3583 | 5956 | 5957 |
| **R-squared** | 0.042 | 0.071 | 0.042 | 0.076 |

*** p<0.01, ** p<0.05, * p<0.1

Robust SEs (clustered at the community level) are in parentheses.

Estimates were weighted using individual sampling weights and adjusted for household and individual responses.

The results presented in this table have been adjusted for the CPI of healthcare service for each province.
